# On the Use of Web Assembly in a Serverless Context

**DOI:** 10.1007/978-3-030-58858-8_15

**Published:** 2020-08-18

**Authors:** Seán Murphy, Leonardas Persaud, William Martini, Bill Bosshard

**Affiliations:** 6 IT University of Copenhagen, Copenhagen, Denmark; 7 University of British Columbia, Vancouver, BC Canada; 8 Zurich University of Applied Science, Winterthur, Switzerland; 9 University of Zurich, Zurich, Switzerland

**Keywords:** Web Assembly, WASM, Serverless, Runtimes

## Abstract

This paper considers how WASM can be run in different serverless contexts. A comparison of different serverside WASM runtime options is considered, specifically focused on wasmer, wasmtime and lucet. Next, different options for running WASM within two serverless platforms – Openwhisk and AWS Lambdai – are compared. Initial results show that a solution which uses the built-in node.js WASM supports is found to work better than using the dedicated WASM runtimes but this has limitations and providing more direct integration with WASM runtimes should be explored further.

## Introduction

*Web Assembly* (WASM) is a technology that has been receiving considerable interest of late. Originally, developed as a portable runtime for browser contexts, its benefits have been recognized for alternative contexts and there is increasing interest in understanding other environments in which it can be used. Serverless is one such context [2–4] and the use of WASM in a serverless context is the focus of this work.

WASM evolved from asm.js^1^ – a previous attempt to define a simple assembly like instruction set which could run efficiently within a browser – and was adopted by the Mozilla foundation in 2017 to co-ordinate development and standardization of the technology across all major browser developers.

WASM is designed to be fast, secure, portable and not tied to any specific language or runtime, although realizing all of these aspects is still something of a work in progress. It is characterized by a simple instruction set which can be formally verified, a stack based Virtual Machine which supports functions and control flow abstractions such as loops and conditionals. A good overview of WASM is provided in [1].

Good support for WASM is provided in today’s major browsers but it is still evolving with significant innovations required to provide support for multi-threaded operation, garbage collection GPU and WebGL supports amongst other items^2^.

As well as browser support, work has been ongoing on developing supports for execution of WASM outside the browser context. Much of this work has been consolidated under the Bytecode Alliance’s work on developing a *Web Assembly Systems Interface* (WASI)^3^ which is a set of APIs available within the WASM runtime which can provide POSIX style capabilities (file system access, network access, process management, etc.). This effort and, in particular, the parallel development effort to create WASI compatible execution engines which can run in different environments is creating new opportunities and use cases for WASM, one of which is serverless.

Serverless solutions today are closely coupled to Docker containers; WASM could provide an alternative or complementary runtime environment which is lightweight, works well with different developer toolchains and could potentially be deployed across different serverless platforms. Having some insight into how this could be realized is the focus of this work.

The remainder of this paper is structured as follows. In Sect.  2 there is a brief comparison of different WASM runtimes which can be used on the server side. This is followed in Sect. 3 by a discussion of different solutions for running WASM within serverless platforms. Finally there is a short conclusion and outlook.

## Evaluation of Serverside WASM in Different Runtimes

Running WASM on the server side requires a means to map from WASM bytecode to native hardware instructions. A number of technologies have been developed to support this: in this work, three were considered – (i) wasmer^4^, (ii), wasmtime^5^ and (iii) lucet^6^.

Both wasmer and wasmtime are runtimes which parse given WASM bytecode, mapping it to native instructions to operate on the host processor using *Just-in-Time* (JIT) compilation mechanisms. The former is under active development by a commercial company, while the latter is developed within the context of a collaborative, standardization activity operated by the Mozilla Foundation. lucet uses a different approach - it performs an *a priori* compilation of the WASM bytecode to produce a standard executable for the host system architecture.

Generating WASM bytecode is generally straightforward although there is some difference in the supports available for different languages and compiler toolchains. Rust and C/C++ currently have the best supports but support for Golang is also good. To compare the different solutions, we chose some reference C algorithm implementations as we knew the code was mature and the toolchain supported generation of WASM output; the Clang toolkit was used to generate WASI compatible WASM binaries^7^ which could be run directly in wasmer and wasmtime and compiled to native with lucet.

**Fig. 1.**
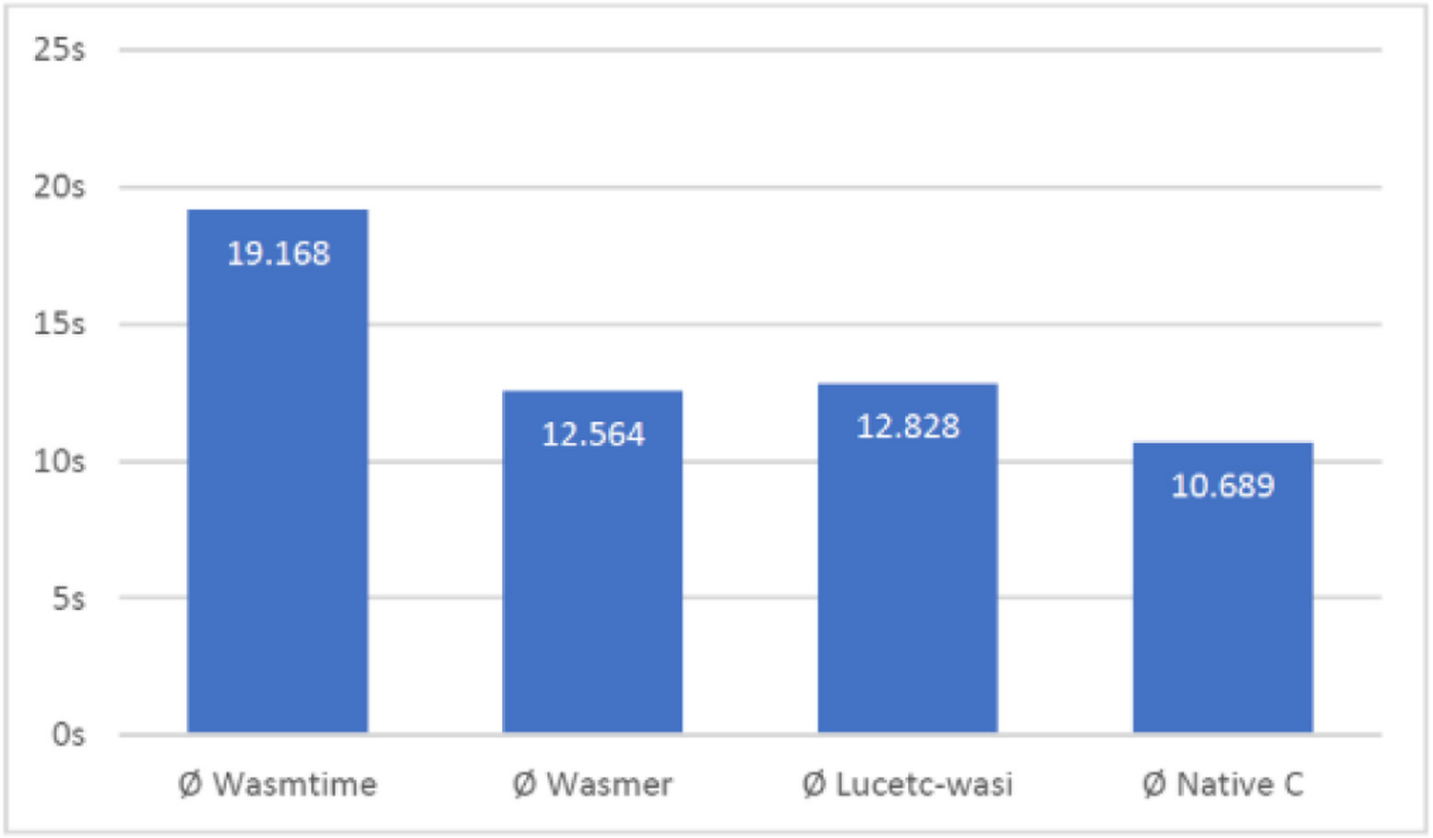
Time taken to determine if 4294967029 is prime

The different approaches were compared using standard memory and compute bound workloads. Figure 1 shows the time taken to determine if a large number is prime using the different approaches.

Here it can be seen that the performance of wasmer is similar to lucet, both performing faster than wasmtime. A number of other comparisons using memory bound and compute bound computation were performed and this conclusion largely held across all the experiments^8^. For this reason, as well as the fact that it has a substantial developer interest, wasmer was chosen as the preferred WASM/WASI runtime^9^. It is also interesting to note that the time difference between the WASM executables and native C is not large, with the WASM executables taking approximately 20% longer than native compiled code.

## Running WASM in Serverless Context

Although the ultimate goal of this work is to understand how WASM can be used as a native runtime in a serverless context, the first priority in this work was to devise a solution which would enable a WASM executable to be run on existing serverless platforms - Openwhisk and AWS Lambda in particular. Hence the initial focus was on the problem of getting WASM code operating in docker container running in different serverless platforms leveraging wasmer as the WASM runtime.

The first approach to address the problem focused on using a Python-flask^10^ wrapper around the WASM executable; the wrapper provided hooks into the serverless platform, offering a small number of HTTP endpoints which the platform could invoke to initialize the serverless function and to trigger it with some input parameters. Although this approach worked in principle, as it used Python, which typically has a large set of dependencies, it resulted in a large container, thus eliminating one of the potential advantages of considering WASM as a runtime.

The second approach was to leverage WASM capabilities within node.js; node.js offers the possibility to run WASM binaries directly within the node runtime (rather than needing a standalone WASM/WASI execution environment). As node.js is well supported in serverless systems, this approach held some promise.

**Fig. 2.**
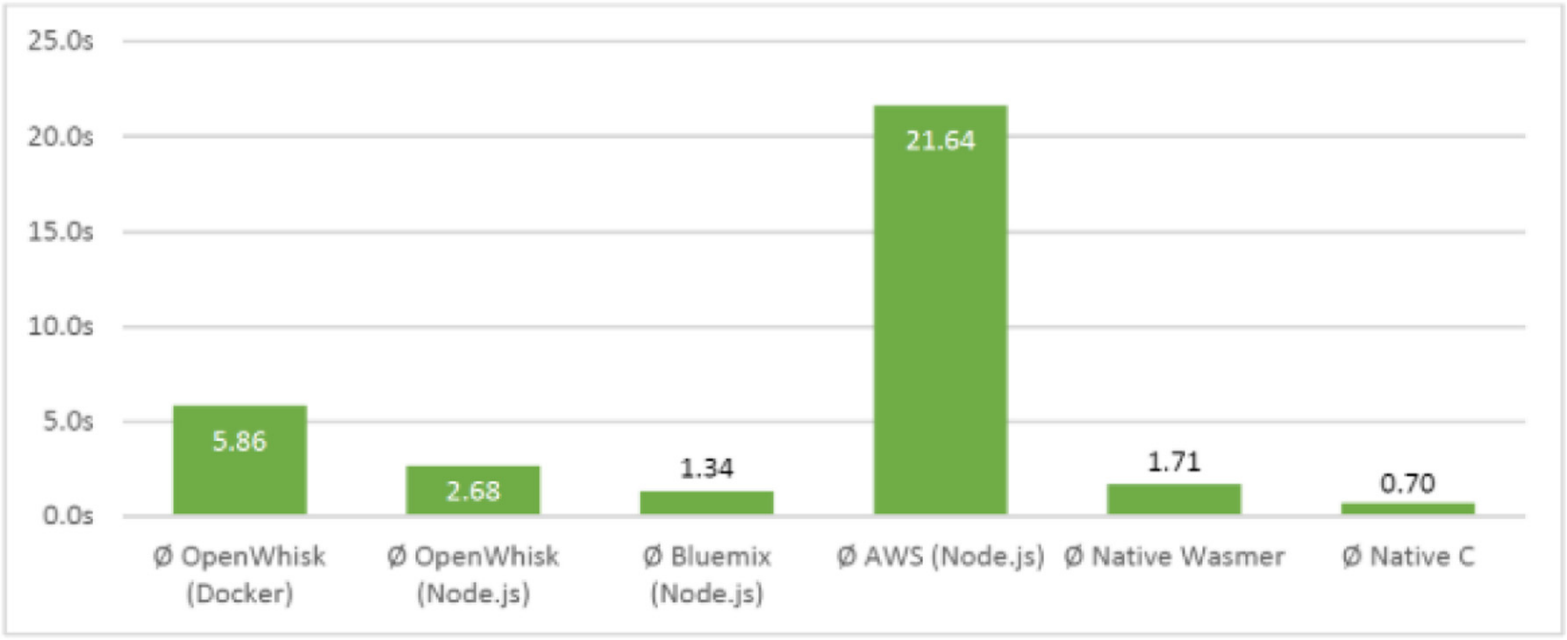
Time taken to calculate the 42^nd^ Fibonacci number

To compare the different solutions a simple WASM file was generated which performed a calculation of the 42^nd^ number in the Fibonacci sequence. The time taken to execute this function when implemented via the Docker/Python solution was compared with that of the node.js solution on different platforms. The results are shown in Fig. 2.

From the figure, it can be seen that using the node.js results in lower latency than the docker solution when run on Openwhisk. One limitation of the node.js solution, however is that only integer types are supported when communicating between the serverless platform and the executable. The slower execution on AWS is still being investigated.

## Conclusion

In this work, the use of WASM in a serverless context was considered: We compared three different options for running server side WASM: wasmer, wasmtime and lucet. wasmer was the best option to proceed with based on the first analysis. Execution of WASM applications within serverless platforms was then considered. One option was to use a container containing glue to the serverless platform, the WASM runtime – wasmer in this case – and the WASM application itself. An alternative approach removed the necessity for the WASM specific runtime as standard node.js engines provide support for running WASM. An initial comparison of these alternatives showed that using node.js can be more efficient than using a dedicated WASM runtime.

Future work will involve deeper comparison of these different approaches for running WASM within different serverless platforms.

## Footnotes

1 http://asmjs.org/.

2 https://www.w3.org/2020/03/webassembly-wg-charter.html.

3 https://github.com/WebAssembly/WASI.

4 https://www.wasmer.io.

5 https://github.com/bytecodealliance/wasmtime.

6 https://github.com/bytecodealliance/lucet.

7 https://depth-first.com/articles/2019/10/16/compiling-c-to-webassembly-and-running-it-without-emscripten/.

8 More details are available in the project report [5] and in the project’s Github repository at https://github.com/WilliamMartini/WASM.

9 The bytecode alliance reached out to note the performance of wasmtime was due to optimizations being disabled by default in previous versions of wasmtime, meaning that similar performance between wasmer and wasmtime can now be expected.

10 https://flask.palletsprojects.com/en/1.1.x/.
